# A novel analytical characterization for short-term plasticity parameters in spiking neural networks

**DOI:** 10.3389/fncom.2014.00148

**Published:** 2014-11-19

**Authors:** Michael J. O'Brien, Corey M. Thibeault, Narayan Srinivasa

**Affiliations:** Center for Neural and Emergent Systems, Information and System Sciences Department, HRL Laboratories LLCMalibu, CA, USA

**Keywords:** STP, recurrent networks, short term plasticity, self-sustaining, neural networks

## Abstract

Short-term plasticity (STP) is a phenomenon that widely occurs in the neocortex with implications for learning and memory. Based on a widely used STP model, we develop an analytical characterization of the STP parameter space to determine the nature of each synapse (facilitating, depressing, or both) in a spiking neural network based on presynaptic firing rate and the corresponding STP parameters. We demonstrate consistency with previous work by leveraging the power of our characterization to replicate the functional volumes that are integral for the previous network stabilization results. We then use our characterization to predict the precise transitional point from the facilitating regime to the depressing regime in a simulated synapse, suggesting *in vitro* experiments to verify the underlying STP model. We conclude the work by integrating our characterization into a framework for finding suitable STP parameters for self-sustaining random, asynchronous activity in a prescribed recurrent spiking neural network. The systematic process resulting from our analytical characterization improves the success rate of finding the requisite parameters for such networks by three orders of magnitude over a random search.

## 1. Introduction

Short-term synaptic plasticity (STP) is a ubiquitous phenomenon occurring throughout the brain. A consequence of synapse physiology, STP produces temporary changes in synaptic efficacy. These facilitating or depressing modulations are driven by the recent synaptic activity—providing a history dependent transmission that may be important for short-term and working memory, as well as cognition. The nonlinear filtering dynamics of STP also appear to play a functional role in cortical gain control (Abbott et al., 1997), burst detection (Lisman, 1997) and temporal integration of information (Maass and Markram, 2002; Haeusler and Maass, 2007; Klampfl and Maass, 2013). Because STP plays a wide reaching role in cortical computation, it is important to understand its precise nature.

Recently, Sussillo et al. (2007) demonstrated that the inclusion of STP to cortical circuits improved network stability. In addition, the authors developed a predictive characterization of that stable regime through control theoretic and computational analysis of the *mean field* model. In this work, we review the work of Sussillo et al. (2007) and further develop the analysis by providing an additional solution to the STP characterization problem, providing further insights into the dynamics induced by STP. Our mathematical derivation is based solely on the underlying commonly used STP model (Markram et al., 1998; Tsodyks et al., 1998; Maass and Markram, 2002), without additional assumptions, and results in an *in vitro* experimentally testable hypothesis. Our characterization illuminates a method for selecting STP parameters to produced specific network properties—something that is useful for recreating empirical results.

We then demonstrate this mathematical characterization in a framework for selecting STP parameters that maintain stable low firing rates in self-sustaining recurrent asynchronous irregular nonlinear (RAIN) activity networks. This topic has been studied before in a variety of settings (Vogels and Abbott, 2005; Kumar et al., 2008; Jayet Bray et al., 2010). However, the biological significance of such activity is open for debate as intact brains are immersed in sensory and functional inputs, but isolated brain tissues have demonstrated such self-sustaining activity in the absence of external inputs (Burns and Webb, 1979; Plenz and Aertsen, 1996; Timofeev et al., 2000). This activity can be explained by endogenously active neurons (Latham et al., 2000a,b), however it is not known if such neurons are required for *in vivo* self-sustaining activity. Despite the uncertainty behind the mechanisms driving RAIN activity, these networks are a useful framework for neural computational systems. For instance, RAIN activity networks are stable to stimulus injections as they do not succumb to network synchronization problems (Vogels and Abbott, 2005), providing an insulating environment for signal transmission. These are also used as the foundation of a RAIN-Entorhinal-Hippocampal model that emulates place cells in rodent navigation (Jayet Bray et al., 2010).

## 2. Methods

All systems used in this work are integrated with a 0.1 ms resolution using the HRLSim^TM^ environment (Minkovich et al., 2014). The models used in this work are described below. The values for the model parameters are given in Table 1.

**Table 1:** **Network parameters used**.

**All networks**	
*C*_m_ = 200 pF	*g*_leak_ = 10 nS
*E*_inh_ = −80 mV	*E*_exc_ = 0 mV
*V*_thresh_ = −54 mV	*V*_reset_ = −60 mV
*V*_rest_ = −74 mV	*R* = 150 MΩ
τ_exc_ = 5 ms	τ_inh_ = 15 ms
*g*^exc^_max_ = 100 nS	*g*^inh^_max_ = 100 nS

### 2.1. Leaky integrate-and-fire neuron model

A leaky integrate-and-fire (LIF) (Abbott, 1999) neuronal model is used in this work, with *excitatory* and *inhibitory* neuron populations. In the LIF model, each neuron, *i*, has spiking behavior governed by the voltage equation

(1) $$\begin{array}{l} C_{m}\frac{dV_{i}}{dt}=g_{\text{leak}}(V_{\text{rest}}-V_{i})+g_{i}^{\text{exc}}(t)(E_{\text{exc}}-V_{i})\\ \;+g_{i}^{\text{inh}}(t)(E_{\text{inh}}-V_{i})+I_{\text{ext}}(t). \end{array}$$

where *V* is the membrane potential; *C_m_* is the membrane capacitance, with input resistance *R*; *I*_ext_ is the externally applied current; *V*_rest_ is the membrane resting potential, *E*_exc_ and *E*_inh_ are the respective excitatory and inhibitory reversal potentials; *g*_leak_ is the leak conductance; and *g*^exc^ and *g*^inh^ are the respective excitatory and inhibitory conductance contributions from neuron *i*'s inputs. A neuronal action potential at time *t*^AP^_*i*_ is modeled by the reset criteria:

(2) $$\underset{t\to t_{i}^{{\text{AP}}^{\text{−}}}}{\lim}V_{i}(t)=V_{\text{thresh}}\text{ and }\underset{t\to t_{i}^{{\text{AP}}^{\text{+}}}}{\lim}V_{i}(t)=V_{\text{reset}},$$

where *V*_thresh_ is the membrane spike threshold and *V*_reset_ is the reset value for the membrane potential following an action potential. After an action potential, the voltage is clamped for a 2 ms refractory period.

The dynamics of the input conductances are modeled as:

(3) $$\tau_{\ell }\frac{dg_{i}^{\ell }}{dt}\text{​}=\text{​}-g_{i}^{\ell }+g_{\text{max}}^{\ell }\sum_{j}W_{ij}\delta (t-t_{j}^{AP})),\text{for }\ell \in \{\text{exc},\text{inh}\}.$$

Here, τ_exc_ and τ_inh_ are the conductance decay constants, δ is the Dirac delta function, *t^AP^_j_* is the time of neuron *j*'s last action potential or spike. The values *W_ij_* indicate the synaptic weight from neuron *j* to neuron *i* at time *t* and are scaled by *g*^exc^_max_ or *g*^inh^_max_ (depending on the type of presynaptic neuron), which are the maximum synaptic conductances. Note that the *W_ij_* are thus dimensionless, and bound to the interval (0, 1).

### 2.2. Short term plasticity

Short term plasticity (STP) temporarily modifies the synaptic weights used in the neural dynamics based on the presynaptic firing rate (Markram et al., 1998; Tsodyks et al., 1998; Maass and Markram, 2002). When invoking STP, the voltage integration assumes an effective synaptic weight, μ_*ij*_, rather than the static synaptic weights *W_ij_*. These effective synaptic weights are short-term modifications of the static weights, dependent on the presynaptic firing rate. This is done by replacing table 1 with

(4) $$\tau_{\ell }\frac{dg_{i}^{\ell }}{dt}\text{​}=\text{​}-g_{i}^{\ell }+g_{\text{max}}^{\ell }\sum_{j}\mu_{ij}(t)\delta (t-t_{j}^{AP})\text{ for }\ell \in \{\text{exc},\text{inh}\},$$

where μ_*ij*_ is modeled as in Tsodyks et al. (1998):

(5) $$\mu_{ij}(t)=A_{ij}\;x_{ij}(t)u_{ij}^{1}(t)$$

(6) $$\;\frac{du_{ij}}{dt}=-\frac{u_{ij}(t)}{\tau_{F_{ij}}}+U_{ij}(1-u_{ij}(t))r_{j}(t)$$

(7) $$\;\frac{dx_{ij}}{dt}=\frac{1-x_{ij}(t)}{\tau_{D_{ij}}}-u_{ij}^{1}(t)x_{ij}(t)r_{j}(t)$$

(8) $$\;u_{ij}^{1}(t)=u_{ij}(t)(1-U_{ij})+U_{ij}.$$

In the above formulation, *A_ij_* is a scaling constant, τ*_D_ij__* and τ*_F_ij__* are the respective depression and facilitation time constants, *u_ij_* tracks synaptic utilization, *x_ij_* tracks synaptic availability, *r_j_* is the instantaneous firing rate of the presynaptic neuron, *U_ij_* is a constant determining the initial release probability of the first spike. *u*^1^_*ij*_ is a term associated with the fraction of opened calcium channels but also serves to simplify the mathematical presentation of the model. For brevity, in our analysis we will use the notation {*A*, *U*, *D*, *F*} in place of {*A_ij_*, *U_ij_*, τ*_D_ij__*, τ*_F_ij__*}.

### 2.3. RAIN networks

Because of the stabilizing effects of STP (Sussillo et al., 2007), we concern ourselves with generating self-sustaining RAIN (SS-RAIN) networks in Section 3.2.3. The architecture and simulation of such networks is described in Sections 2.3.1 and 2.3.2. In Section 2.3.3, we find static weight parameters for SS-RAIN that yield an average network spike rate of 12 Hz, which will be used for the basis of the rest of the work presented.

#### 2.3.1. Architecture

**Figure 1:**
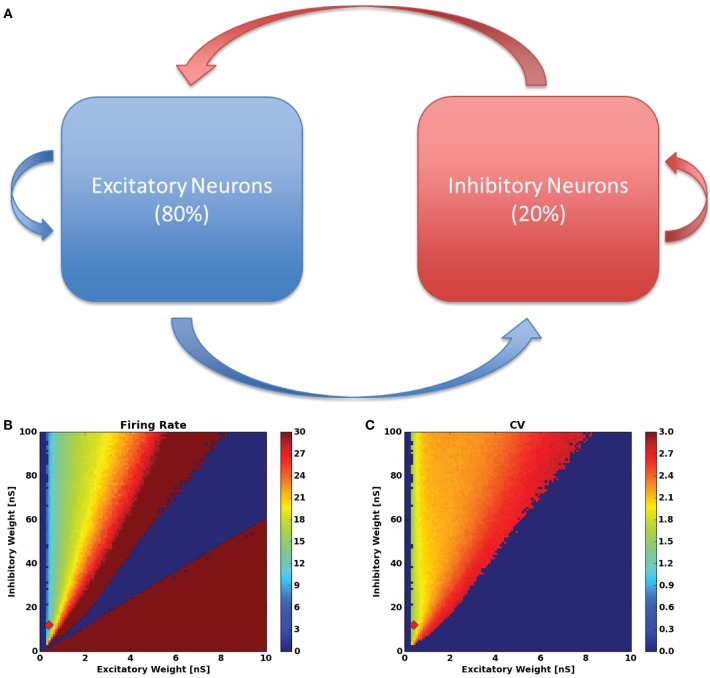
**(A)** RAIN network configuration. The red arrows indicate inhibitory connections and the blue arrows are excitatory connections. **(B)** The firing rate and **(C)** inter-spike-interval coefficient of variation for the networks tested in the synaptic weight parameter sweep. The red diamond in **(B,C)** indicates the parameters used throughout the rest of this work.

To build an SS-RAIN network, we construct a sparsely connected 80/20, network using 8000 excitatory neurons and 2000 inhibitory neurons with a connectivity of 1.5%, meaning that for any two neurons *j* and *i*, the probability that there is a connecting synapse from *j* to *i* is 1.5%. Figure 1A is the resulting network diagram. The synaptic strengths from the excitatory population are uniform and denoted by *W*_exc_ while the uniform synaptic weights from the inhibitory pool are *W*_inh_.

#### 2.3.2. RAIN simulation

The RAIN network simulation is conducted as follow: 200 random neurons (allowing both excitatory and inhibitory) are stimulated with Poisson distributed spikes for 50 ms, resulting in an initial activity burst within the network. After the initial 50 ms of spike injection, all external inputs are turned off and the network's activity is recorded for 2 s. The final 100 ms of activity is analyzed for average firing rate and inter-spike-interval coefficient of variation for each neuron (this avoids a transient period from the initialization stage). These values are then averaged across the network, and recorded. The goal is to find sustainable asynchronous network activity between firing rates *r*_min_ and *r*_max_ with a coefficient of variation above 1.

#### 2.3.3. Static weight search

With the network parameters in Table 1, we seek to find static weights that produce SS-RAIN. We proceed by performing a parameter sweep across the values (*W*_exc_, *W*_inh_) ∈ (0, .1) × (0, 1) with a discretization of 0.001 and 0.01 in each dimension respectively, resulting in roughly 10,000 combinations. For each parameter set, we simulate the network as in Section 2.3.2, with *r*^min^ = 10 Hz and *r*^max^ = 20 Hz. With this approach, we were able to find parameters with a low firing rate and asynchronous activity, all sustained for at least 2 s. The results of the parameter search are shown in Figures 1B,C. Throughout the rest of this work, we used the static weights (*W*_exc_, *W*_inh_) = (0.04, 0.12). This procedure is similar to that used in Vogels and Abbott (2005) for finding a RAIN network.

### 2.4. Searching the STP parameter space

In Section 3.1.3 we derive an STP characterization which yields a transitional point in synaptic dynamics which we call the *critical firing rate*, or *r*_crit_. In Section 3.2.3 we guide an STP parameter search through leveraging our characterization to partition the space. For this, we restrict the STP parameters such that *U* ∈ (0, 1) as it represents a probability, and τ_*D*_, τ_*F*_ ∈ (0, 1), as in Sussillo et al. (2007). We label the synaptic connection types as EE, EI, IE, and II for excitatory-to-excitatory, excitatory-to-inhibitory, inhibitory-to-excitatory and inhibitory-to-inhibitory, respectively.

We proceed by defining different dynamic synapse regimes, characterized by *r*_crit_, and explore each regime. For each connection type (EE, EI, IE, and II), we independently consider an STP parameter class of N, D, T, A, B, or G, defined by *r*_crit_ in Table 2 (Section 3.2.3), giving us 6^4^ = 1296 different network types defined by the STP parameter combinations for the four different connection types. Specifically, we define a network signature to be WXYZ, for (W, X, Y, Z) ∈ {N, D, T, A, B, G}^4^, which corresponds to a network where the EE, EI, IE, and II connections are governed by the W, X, Y, and Z class of STP parameters, respectively. For example, if WXYZ = DTNB, then the STP parameters for the EE connections have 0 Hz < *r*_crit_ ≤ 4 Hz, the EI connections have 4 Hz < *r*_crit_ ≤ 8 Hz, and so on. For each of the 1296 network types, WXYZ, the STP parameters for the respective synapse types are selected from the corresponding STP parameter regime uniformly at random (with the caveat discussed in the following paragraph). For each network configuration type WXYZ, we chose 10^6^ sets of parameters to test, uniformly at random from the subspace defined by WXYZ.

**Table 2:** **The STP parameter space was partitioned with respect to the resulting value of *r*_crit_, where the boundaries were chosen to be the common brain rhythm boundaries (Buzsáki, 2006)**.

**Key**	**Rhythm**	**Range (Hz)**
N	*Null*	*r*_crit_ ≤ 0
D	δ	0 < *r*_crit_ ≤ 4
T	θ	4 < *r*_crit_ ≤ 8
A	α	8 < *r*_crit_ ≤ 12
B	β	12 < *r*_crit_ ≤ 30
G	γ	30 < *r*_crit_

*Note that N corresponds to the null regime, as negative presynaptic firing rates do not exist. However, it makes sense to consider r_crit_ in this regime since this implies a synapse characterized by depressing for all presynaptic firing rates. For each of these parameter types, the synapse is facilitating when the steady-state presynaptic firing rate r is less than r_crit_, and depressing whenever r > r_crit_. In particular, synapses of type N are always depressing*.

As discussed above, we selected the parameters uniformly at random over the WXYZ-type restricted space. However, we imposed an additional *small d*μ constraint on the parameters allowed. That is, we required the dynamical synapses to be slow moving. Specifically, |*d*μ| < .01 is the constraint on the derivative of the dynamic synapse we used. We found that this additional requirement yields moderate increases to our experiment success rates in preliminary tests. We note that *d*μ was assumed to be small in the analysis conducted in Section 3.1.2, which suggested the constraint. We discarded approximately 18.8% of the 10^8^ randomly picked STP parameter sets because they violated this constraint.

## 3. Results

### 3.1. An analytical characterization of STP

The introduction of STP into the neural dynamics of a network can, to name a few benefits, influence the stability of the RAIN activity via self-tuning control (Sussillo et al., 2007), improve reinforcement learning (O'Brien and Srinivasa, 2013) and improve a network's ability to propagate signals (O'Brien, 2013). In this work, we will expand upon Sussillo et al. (2007) where it is demonstrated that synaptic STP dynamics can support a fixed average network firing rate through self-tuning. Based on Sussillo et al. (2007), we present the fixed-point heuristics in Section 3.1.1, leading to the in-depth analysis conducted in Section 3.1.2. In Section 3.1.2 the linear approximation to the change in firing rate with respect to the change in network inputs is presented with the mathematical derivation using mean field theory fully developed in Appendix A. In Section 3.1.3 we derive a novel characterization of of the STP parameter space, distinguishing between facilitating and depressing synapses.

#### 3.1.1. Steady state STP

To begin, we consider the steady state for Equations (5) to (8), given a steady firing rate r^*^, where steady state values are indicated by asterisks. As noted in Section 2.2, we use {*A*, *U*, *D*, *F*}:

(9) $$\;\mu_{ij}^{*}=A_{ij}x_{ij}^{*}U_{ij}^{1^{*}}$$

(10) $$\;u_{ij}^{*}=\frac{F_{ij}U_{ij}r_{j}^{*}}{1+F_{ij}U_{ij}r_{j}^{*}}$$

(11) $$\;x_{ij}^{*}=\frac{1}{1+D_{ij}U_{ij}^{1^{*}}r_{j}^{*}}$$

(12) $$U_{ij}^{1^{*}}=u_{ij}^{*}(1-U_{ij})+U_{ij}.$$

Assuming that the static weights *W_ij_* were selected to give an average network firing rate at r^*^, the dynamical synapses can produce a fixed point at firing rate r^*^ if the multiplicative constants *A_ij_* are selected in the following way. For a given set of STP parameters and desired firing rate r^*^, pick

(13) $$A_{ij}(U_{ij},D_{ij},F_{ij},\text{​}r^{*})\text{​}=\text{​}\frac{W_{ij}}{x_{ij}^{*}(U_{ij},D_{ij},F_{ij},r^{*})U_{ij}^{1^{*}}(U_{ij},D_{ij},F_{ij},r^{*})}.$$

We can now compute the effective weight μ_*ij*_ at r^*^_j_ = r^*^:

(14) $$\mu_{ij}=A_{ij}x_{ij}^{*}U_{ij}^{1^{*}}=\frac{W_{ij}}{x_{ij}^{*}U_{ij}^{1^{*}}}x_{ij}^{*}U_{ij}^{1^{*}}=W_{ij},$$

which yields fixed firing dynamics, by assumption. Thus, with *A_ij_* picked in this way, we attain a fixed-point firing rate for the system.

We now consider a heuristic for the stability of r^*^. In order to make r^*^ a stable fixed point, Sussillo et al. (2007) proposed that for a given firing rate *r*, the effective synaptic weights μ_*ij*_ should behave as follows.

1. When *r* < r^*^:
  - Increase synaptic strength efficacy for *E* → *E* and *I* → *I* synapses,
  - Decrease synaptic strength efficacy for *E* → *I* and *I* → *E* synapses.
2. When *r* > r^*^:
  - Decrease synaptic strength efficacy for *E* → *E* and *I* → *I* synapses,
  - Increase synaptic strength efficacy for *E* → *I* and *I* → *E* synapses.

**Figure 2:**
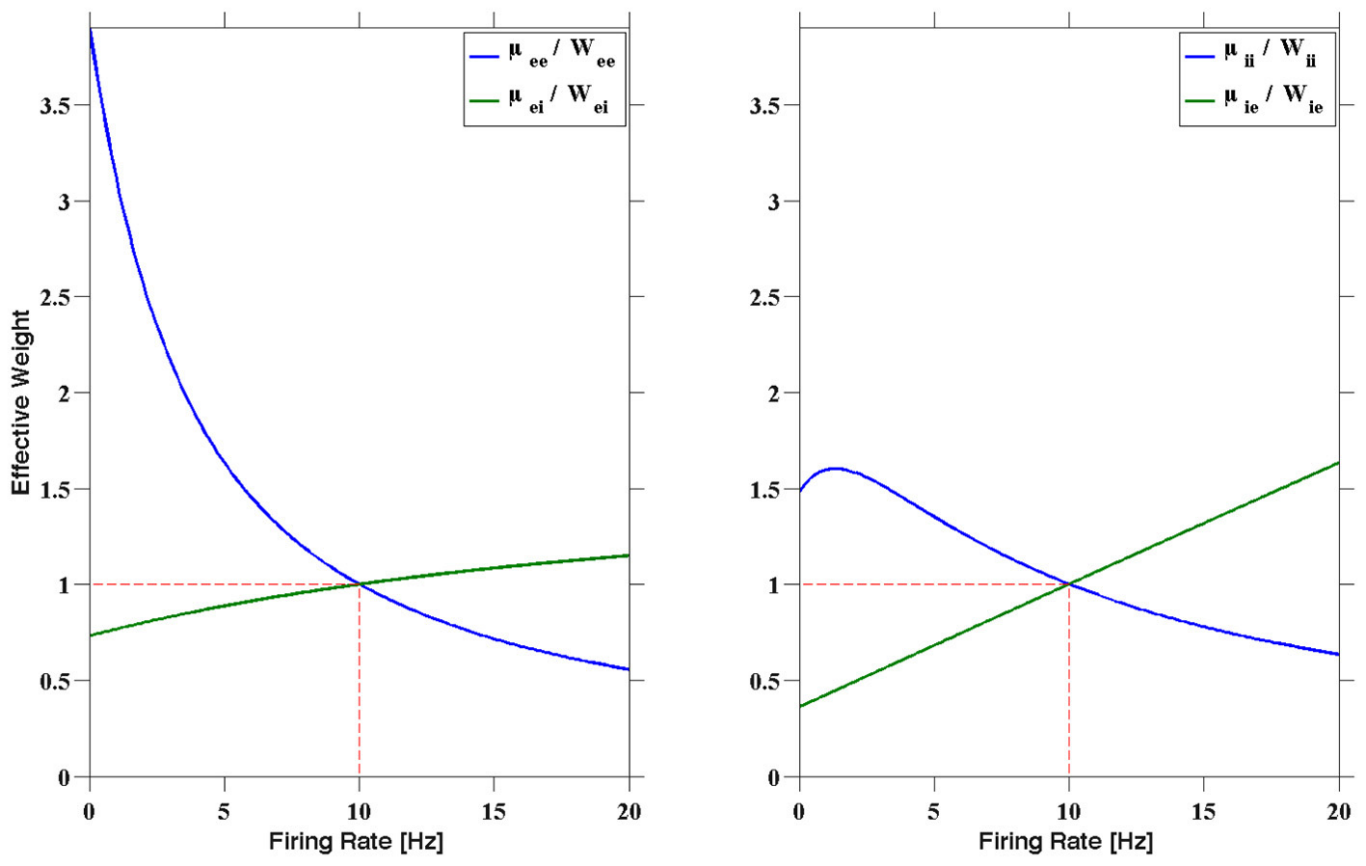
**The normalized dynamic synapses plotted as a function of the presynaptic firing rate**. The STP parameters can be chosen to produce a fixed point firing rate. The parameters used here are the *R1* parameters presented in Sussillo et al. (2007), with fixed point r^*^ = 10 Hz. Note that at 10 Hz we have μ_mn_ = *W_mn_*, where *W_mn_* was chosen to produce stable RAIN activity, for *m*, *n* ∈ {*e*, *i*}.

This heuristic is visualized in Figure 2. In the left figure, for instance, the normalized excitatory to excitatory and inhibitory to excitatory dynamic synapses (μ_*ee*_/*W_ee_* and μ_*ei*_/*W_ei_* respectively) are shown as a function of the presynaptic firing rate. The corresponding effects on a postsynaptic excitatory neuron can be deduced from this. When a postsynaptic excitatory neuron is firing greater than 10 Hz, then the assumption is that the entire network, including the presynaptic neurons, is also firing at a rate greater than 10 Hz. In this case, then μ_*ee*_ < *W_ee_* and μ_*ei*_ > *W_ei_*, providing less excitation, as well as more inhibition, than the static synapses would, resulting in a slowing of the network firing. In contrast, if an excitatory postsynaptic neuron is firing slower than 10 Hz, then μ_*ee*_ > *W_ee_* and μ_*ei*_ < *W_ei_*, providing more excitation and less inhibition to speed up the postsynaptic neuron, and in turn, the network. A similar argument can be made for the II and IE curves on the right.

#### 3.1.2. Linear first order dynamics

Using linear perturbation theory, Sussillo et al. (2007) demonstrated an expression for the first order change in the excitatory firing rate δ*r_e_* with respect to a small perturbation in the excitatory and inhibitory input currents δ*v_e_* and δ*_i_* respectively. A detailed exposition of the derivation is given in Appendix A. A network of an excitatory population and an inhibitory population is assumed, in agreement with the neural networks used in this manuscript. The resulting expressions are

(15) $$\begin{array}{l} \frac{\delta r_{e}}{\delta v_{e}}\approx \frac{\beta_{e}(1+\beta_{i}n_{i}{\bar{W}}_{ii})}{\alpha }[1+d_{ee}\frac{\beta_{e}n_{e}r_{e}^{*}(1+\beta_{i}n_{i}{\bar{W}}_{ii})}{\alpha }\\ \;-d_{ie}\frac{\beta_{e}\beta_{i}n_{i}n_{e}r_{e}^{*}{\bar{W}}_{ei}}{\alpha }\\ \;-d_{ei}\frac{\beta_{i}\beta_{e}n_{e}n_{i}r_{i}^{*}{\bar{W}}_{ie}}{\alpha }\\ \;+d_{ii}\frac{\beta_{i}^{2}\beta_{e}n_{i}^{2}n_{e}r_{i}^{*}{\bar{W}}_{ei}{\bar{W}}_{ie}}{\alpha (1+\beta_{i}n_{i}{\bar{W}}_{ii})}], \end{array}$$

and

(16) $$\begin{array}{l} \frac{\delta r_{e}}{\delta v_{i}}\approx -\frac{\beta_{i}\beta_{e}n_{i}{\bar{W}}_{ei}}{\alpha }[1+d_{ee}\frac{\beta_{e}n_{e}r_{e}^{*}(1+\beta_{i}n_{i}{\bar{W}}_{ii})}{\alpha }\\ \;-d_{ii}\frac{\beta_{i}n_{i}r_{i}^{*}(1-\beta_{e}n_{e}{\bar{W}}_{ee})}{\alpha }\\ \;-d_{ie}\frac{\beta_{e}\beta_{i}n_{i}n_{e}r_{e}^{*}{\bar{W}}_{ei}}{\alpha }\\ \;+d_{ei}\frac{r_{i}^{*}(1+\beta_{i}n_{i}{\bar{W}}_{ii})(1-\beta_{e}n_{e}{\bar{W}}_{ee})}{\alpha {\bar{W}}_{ei}}], \end{array}$$

where $d_{mn}:=\frac{d{\bar{\mu }}_{mn}}{dr_{n}^{*}}$ for {*m*, *n*} ∈ {*e* = *exc*, *i* = *inh*} are derivatives of the mean dynamic synaptic weight from neuron *n* to neuron *m* with respect to a change in the steady state firing rate r^*^ of neuron *n*. The rest of the values in Equations (15) and (16) are positive constants as defined in Table 3.

**Table 3:** **STP linear perturbation constants for neural populations {*m*, *n*} ∈ {*e* = *exc*, *i* = *inh*}**.

β_*m*_ .................................derivative of the rate transfer function
*W_mn_* ............... mean static synaptic weights from population *n* to *m*
*n_m_* ....................number of presynaptic neurons from population *m*
r^*^_m_ .........................mean steady state firing rate for population *m*
α .......................................................positive constant

*See Appendix A. for more details*.

As all the constants are positive, Equations (15) and (16) yield net changes dependent on the signs of the derivatives *d_mn_*. For example, consider a situation in which it is desirable to minimize the increase in excitatory firing rate despite an increase in excitatory input. In this case, it is desirable to make $\frac{\delta r_{e}}{\delta v_{e}}$ small, which can be done by restricting *d_ee_* and *d_ii_* to be negative, and *d_ei_* and *d_ie_* to be positive per Equation (15).

Note that the implications of Equation (16) are less clear as the signs of the terms also depend on the parity of 1 − β*_e_n_e_W_ee_*. The correct parity choice can be determined for specific situations, as done in Sussillo et al.(2007). Since the sign parity of *d*μ is so fundamental to the dynamics, we will further examine it in the next section.

#### 3.1.3. Critical firing rate

Given that the dynamics of the network depends on the rates of change of the dynamic synapses, we will proceed to characterize the parameters that give the desired characteristics. As before, let *m*, *n* ∈ {exc, inh}. As *d_mn_* = *d*μ^*^_mn_/dr^*^, we begin by computing $\frac{d\mu }{dr}$ from Equations (9) to (12), where, as noted in Section 2.2, we use {*U*, *D*, *F*}. We get:

(17) $$\frac{d\mu }{dr}=\frac{U(F-DF^{2}Ur^{2}-2DFUr-FU-DU)}{{\left(DFUr^{2}+DUr+FUr+1\right)}^{2}}.$$

As *U* is a probability, and thus positive, the sign of the derivative is completely determined by the expression

(18) $$F-DF^{2}Ur^{2}-2DFUr-FU-DU,$$

which is quadratic in *r*. To find the transition point in Equation (17), we equate Equation (18), to zero and solve for *r*. Recalling that *U* is bound to (0, 1), we arrive at

(19) $$r_{\text{crit}}=-\frac{1}{F}+\sqrt{\frac{1-U}{UDF}},$$

where we have taken the (potentially) positive branch of the solution since negative firing rates do not make sense. We note that Equation (18) is dominated by a negative quadratic coefficient, so as the steady-state firing rate *r* grows large, the synapse governed by Equation (17) is depressive. Thus, for any set of parameters, {*U*, *D*, *F*}, commonly referred to as *UDF*, one of two cases can happen. First, if *r*_crit_, as computed by Equation (19), is negative, the synapse governed by the *UDF* parameters will always be depressive. On the other hand, for a positive *r*_crit_, then for a steady-state firing rate *r* < *r*_crit_, the synapses governed by *UDF* will be facilitating, whereas for *r* > *r*_crit_, the *UDF* synapses will be depressive. In Section 3.2.3, it is with this synaptic characterization that we search the *UDF* parameter space for STP parameters to give SS-RAIN networks.

### 3.2. Simulation experiments

#### 3.2.1. Partitioning the STP parameter space

**Figure 3:**
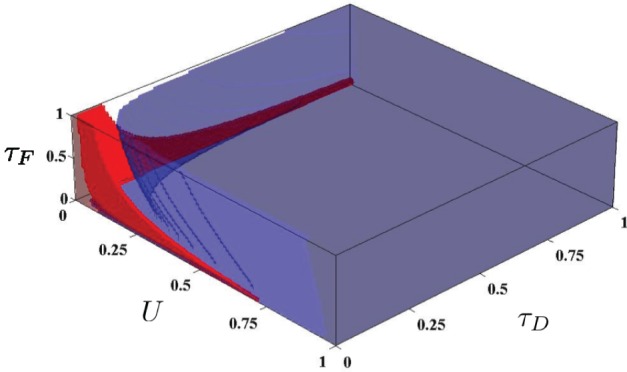
**The N and P volumes from Sussillo et al.(2007) as generated using the *r*_crit_ characterization**. Red is the P volume and blue is the N volume.

In Sussillo et al.(2007), the authors generate simple volumes within the STP parameter space, which they denote the P volume and the N volume. The authors define P and N volumes as the regions in which the STP parameters produce facilitating and depressing synapses, respectively, for all steady state firing rates between 10 and 100 Hz. The P and N volumes are used for generating the required synapse types used in Sussillo et al.(2007) for producing steady state attractor dynamics in the SNN. In order to generate these volumes, the parameter space was discretized and sampled as (*U*, *D*, *F*) ∈ (0, 1)^3^ (where *D* and *F* are in seconds) with a discretization of 0.014 in each dimension. For each point, the STP dynamics were checked to determine if the parameters produced facilitating synapses or depressing synapses. This discretization implies the checking of at least 357,911 parameter combinations, each of which was checked for producing strictly facilitating or depressing dynamics for some discretization of the entire range of firing from 10–100 Hz. However, *r*_crit_, derived above, directly yield the boundary of these volumes by setting *r*_crit_ to 10 or 100 Hz and solving. The volumes are shown in Figure 3, produced with minimal effort.

#### 3.2.2. A dual purpose synapse

**Figure 4:**
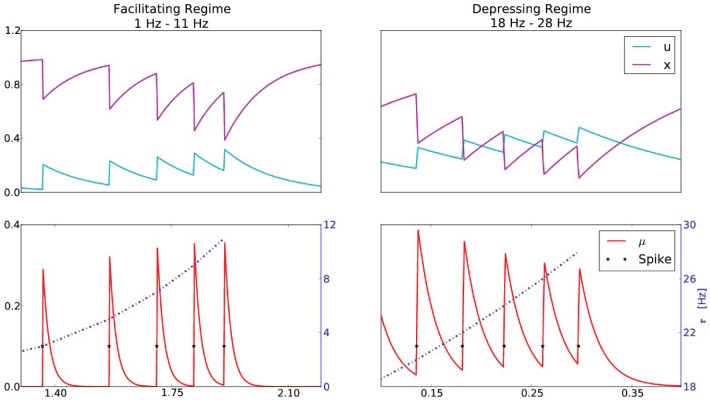
**A synapse that exhibits both facilitation and depression**. The STP parameters are *U* = 0.1, τ_*D*_ = 120 ms, τ_*F*_ = 150 ms, yielding *r*^crit^ = 15.7 Hz. The top series are the STP variables *u* and *x*, and the bottom series is the resulting synaptic efficacy μ at the corresponding presynaptic firing rates *r*. The left column corresponds to a presynaptic firing rate that increases from 1 to 11 Hz (incremented by 2 Hz), which is below *r*_crit_, manifesting in a facilitating synapse (0 < *d*μ). The right column corresponds a presynaptic firing rate that increases from 18 to 28 Hz (incremented by 2 Hz), above *r*_crit_, manifesting in a depressing synapse (*d*μ < 0).

We also find that many synapses have two functional regimes, based on the presynaptic firing rate and the value of *r*_crit_. In particular, any synapse that has strictly positive *r*_crit_ can in theory be both facilitating and depressing. This is demonstrated in Figure 4, where the presynaptic firing rate is varied and the resulting synaptic efficacies are plotted. The STP parameters are chosen so that *r*_crit_ ≈ 15.7. Thus, for presynaptic firing rates *r* < *r*_crit_, the synapse is facilitating, whereas for *r* > *r*_crit_ the synapse is depressing.

This simple demonstration suggests an experimental paradigm to verify the underlying model. It was demonstrated in Wang et al.(2006) that a synapse can be both facilitating and depressing. Furthermore, the authors observed that depression becomes more apparent as the presynaptic firing rate increases. These results are in agreement with our analysis, however a further exploration could be conducted to verify the agreement of the transition point with Equation (19). After estimating the parameters *U*, *D* and *F*, one could compute *r*_crit_ and demonstrate that for presynaptic firing rates below *r*_crit_, facilitation is dominant and that as the presynaptic firing rate increases past *r*_crit_, depression begins to dominate.

#### 3.2.3. Finding STP parameters for SS-RAIN

In Section 3.1.1 it was conjectured that the derivative of μ (referred to in this section as *d*μ) is important to the dynamics induced by STP, and Section 3.1.2 and 3.1.3 provide analytical arguments in support of this conjecture. In addition, in Sussillo et al.(2007), the authors propose which derivative signs are important for a fixed point firing rate with respect to their mean field current injection model. Despite the accomplishments of Sussillo et al.(2007), the general case would have experimental goals and assumptions which vary from those in Sussillo et al.(2007), requiring more general techniques for generating *good* parameter sets that lead to the desired dynamics. For instance, the analysis done in Section 3.1.2 assumes small changes in network inputs, which will not always be true. Furthermore, the analysis is a first order approximation to the network dynamics, ignoring the nonlinear dynamical interactions. The higher order dynamics are of course very difficult to predict, and it is currently unclear if any concrete conclusions can be drawn. The first order conclusions, however, do indicate that the sign parity of *d*μ is an important parameter, and the general analytical result derived in Section 3.1.3 is a powerful tool that characterizes the sign parity regions. We leverage the tool as described below.

We have established that different types of STP parameters can generate different desirable network dynamics. For instance, with the proper STP dynamics in place, RAIN networks would be much less sensitive to network fluctuations, due to the emergent stabilization effects demonstrated in Sussillo et al.(2007). As a simple application of our STP characterization, in this section we attempt to classify regions of the STP parameter domain with respect to their likelihood of producing the dynamics requisite for the specific SS-RAIN networks described in Section 2.3. For our purposes we only require sustained activity at a reasonable rate, for a few seconds, as defined below. We use r^*^ = 12 Hz, which is at the boundary of the α and β rhythms (see Table 2), as our desired firing rate for setting the value of *A_ij_* in Equation (13) because this is the steady state firing rate that naturally arises from our selected static weights in the RAIN experiment of Section 2.3.3. Note that because STP has three parameters, and each network has four connection types, if we choose the STP parameters at random and independently for the different connection types, we are choosing parameters from a 12 dimensional space, making a brute force search intractable with typical laboratory computing resources. Furthermore, as it turns out, a random search is unlikely to yield acceptable parameters, as demonstrated by the first entry in Table 4.

**Table 4:** **The success rate for finding STP parameters generating SS-RAIN networks for various regions of the STP parameter domain**.

**Type**	**# Successes (*S*)**	**% Success**	**×RAND (*S*/*S_r_*)**
RAND	*S_r_*: = 9	0.0009	1
GGGT	24,461	2.4461	2718
GGGA	23,234	2.3234	2582
GBGT	21,505	2.1505	2389
GGGB	20,949	2.0949	2328
GGGG	19,521	1.9521	2169
GBGA	15,830	1.583	1759
GBGD	15,586	1.5586	1732
GGDN	15,141	1.5141	1682
GGNN	13,413	1.3413	1490
BBGG	12,664	1.2664	1407
GGDD	12,147	1.2147	1350
GBGB	10,794	1.0794	1199
BBGB	10,514	1.0514	1168
BBGT	10,445	1.0445	1161
GBGN	9819	0.9819	1091
BBGA	9595	0.9595	1066
GGBT	8975	0.8975	997
BGGG	8957	0.8957	995
GGBA	8251	0.8251	917
GGGD	8048	0.8048	894
BAGT	7570	0.757	841
BAGA	7327	0.7327	814
GAGT	7200	0.72	800
BAGB	7048	0.7048	783
GAGD	6825	0.6825	758
BAGG	6404	0.6404	712
GBGG	5782	0.5782	642
GAGN	5612	0.5612	624
GGND	5391	0.5391	599
GGAT	5375	0.5375	597
GGBB	5315	0.5315	591
BTGT	4959	0.4959	551
BTGA	4950	0.495	550
BBGD	4904	0.4904	545
BTGB	4674	0.4674	519
ABGG	4641	0.4641	516
GAGA	4568	0.4568	508

*Uniformly at random is the first entry, with the number of successes *S*^r^ = *9*, and the most prolific regions defined in Section 3.2.3 follow. The third column gives the success ratio greater than RAND*.

To improve on searching at random, we used the results derived in Section 3.1.3 to partition the STP parameter space. Specifically, we consider the STP parameter regimes defined in Table 2, conditioned on *r*_crit_. Using the partition in Table 2, we define the network types to be explored as discussed in Section 2.4, resulting in network signatures WXYZ. For each network signature, we construct 10^6^ instances of the network type, as in Section 2.3.1, with the STP parameters drawn independently and uniformly at random from the restricted parameter space (see Section 2.4 for details), and simulate the resulting network, as in Section 2.3.2, with target firing rates of *r*^min^ = 1 Hz and *r*^max^ = 50 Hz. A success was recorded if at the end of 2 s, the network was firing at a rate within the target zone, as described in Section 2.3.2. Note that though the success range was defined to be 1–50 Hz, almost all of the successes were from the interval 10–30 Hz. We present the highest percentages of success in Table 4. We also present, as the first entry of the table, the experiment with the RAND signature, which consists of the results for networks with parameters chosen uniformly at random without domain restriction. In the RAND case, 16 separate trials of 10^6^ simulations were done while varying the random number generator seed and changing the set of STP parameters used just to ensure reliability of results. The best number is reported, though from the 16 trials, the average number of successes was 4.69, with a minimum of 1 success.

Though the success rate for the most successful region in Table 4 is only around 2.4%, it is three orders of magnitude larger than the success rate for searching at random. This provides a preliminary boundary for which regions of the STP parameter domain can be explored for SS-RAIN spiking activity networks. From this analysis, it is clear that the ideal parameter region requires that the excitatory synapses (EE and EI) have an *r*_crit_ value (and therefore the facilitation-depression transitional point) above 30 Hz in many of the best configurations, and above 12 Hz in the best 20 configurations. Both values are at or above the target firing rate of 12 Hz. Conclusions from the other two types of synapses are not as clear.

## 4. Discussion

### 4.1. STP characterization

Though this work is guided by the analysis of Sussillo et al.(2007), our mathematical derivation of *r*_crit_ is independent of the assumptions that are important in the linear perturbation techniques. The result should be generally applicable despite varying conditions of the underlying networks, which may or may not fall in line with the assumptions used in the perturbation analysis. The generality of the result is because the analytical derivation does not depend on the underlying neural model, connectivity or scale. The only assumption made in the derivation is that of the underlying STP model used, which is the commonly referenced model of Tsodyks et al.(1998). The analytical conclusions that we draw here should therefore be applicable to different neuron models such as the more robust Izhikevich neuron model (Izhikevich, 2003, 2004) as well as rate based models.

Furthermore, the value of *r*_crit_ provides a deeper understanding of the synaptic dynamics involved, yielding heuristics for picking synaptic parameters that manifest in particular behaviors. For instance, the propagation of signal across a subcircuit of neurons could be boosted by using synapses that have facilitation properties within a certain firing regime. Simultaneously, reverberations can be minimized if the same synapses manifest in depressing dynamics at prescribed firing rates deemed too high. These network traits can be realized by picking *r*_crit_ properly, which can be done as demonstrated in Section 3.2.2.

In using *r*_crit_ to partition the STP parameter space, we found that requiring particular ranges of *r*_crit_ for the different synapse types greatly increases the chance of finding STP parameters that result in SS-RAIN networks. The particular results we found in Section 3.2.3 will vary with different network types, just as the specifications given in Sussillo et al.(2007) are not generally applicable, but the prescribed method of exploring the space with respect to *r*_crit_ is generally applicable and yields interesting information about the underlying networks. If knowledge is known *a priori* about regimes that should likely be facilitating or depressing, requirements can be placed on *r*_crit_ to greatly prune the search space, despite other assumptions. Experiments of greatly differing assumptions and requirements can exploit the STP characterization to find networks with the proper dynamics.

The search for SS-RAIN networks is a simple demonstration of the usefulness of the developed STP characterization. However, this does not *experimentally* demonstrate the general applicability of the STP characterization. We have reason to believe that the characterization is widely useful in practice because of the few assumptions required for the analytical derivation and based on our other studies. Specifically, STP was used in previous work to stabilize RAIN dynamics in networks of various scales (O'Brien and Srinivasa, 2013). In that work, STP was shown to increase the stability of RAIN dynamics in networks of between the sizes 100 and 10,000 neurons, though the focus of the study was different and the networks were not required to be self-sustaining. This suggests the usefulness in applying properly tuned STP dynamics in networks of scale. Furthermore, we are preparing a manuscript based on the work in O'Brien (2013) that specifically uses the STP characterization derived here to pick STP parameters that regulate signal propagation.

Our results and the results of Sussillo et al.(2007) are insightful, but further work in this area is necessary to develop a complete understanding of the role STP plays in neural dynamics. However, analysis and simulations are often conducted in a vacuum, without strong links to the experimental world. Through our mathematical analysis, we have provided an experimentally verifiable prediction to ground our work, with hopes that our prediction will lead to further refinements of the underlying STP model, if necessary.

### 4.2. Bootstrapping success

In the simple application of the STP characterization, we found that for the specific SNN described in Section 2.3 selecting the *r*_crit_ values from the γ-rhythm regime for E→E, E→I, and I→E connections, and *r*_crit_ from the θ-rhythm regime for the I→I connections greatly improves (over random selection) the success rate of finding STP parameters that yield SS-RAIN activity in our networks, the success rate remains low at 2.4%. This is a result of the severe manner in which the network was initialized, with an abrupt jump-start. This initialization method was chosen in order to make the property of self-sustaining extremely difficult to achieve. As a result, the parameters that were found achieving success were very robust to network fluctuations. In contrast, Kumar et al.(2008) provides external current to the network until the neurons are all firing at a pre-determined firing fixed point, after which the inputs were slowly removed. They found that abruptly removing the network inputs produces strong network transients that compromise stability. Our method of jump-starting the network by bombarding it with inputs for 50 ms, and then abruptly removing the inputs without checking if the network has stabilized at some acceptable firing rate is far from the gentle jump-start Kumar et al.(2008) uses, yet we are able to find networks that perform very well by exhibiting self-sustaining behavior. The sustained spiking in our networks lasts much longer than the networks reported in Kumar et al.(2008). This can be expected because of the stabilizing effects rendered by STP (Sussillo et al., 2007). However, it is likely that using a gentle jump-start method, such as that proposed in Kumar et al.(2008), would boost the success rates of our SS-RAIN network experiments.

Another method for boosting the success rate would be to bootstrap our STP methodologies with other synaptic plasticity rules. Spike-timing dependent plasticity (STDP) (Markram et al., 1997; Bi and Poo, 1998; Song et al., 2000; Song and Abbott, 2001) could be used in temporally longer experiments to slowly evolve the underlying static weights until the network can remain stably active. Homeostatic plasticity (Turrigiano and Nelson, 2004; Yeung et al., 2004) could also be a candidate to stabilize network dynamics in a similar nature to STP. Homeostatic plasticity modulates the synaptic efficacies based on the postsynaptic neuron's firing rate, playing a similar role that STP plays with respect to presynaptic firing rates.

### 4.3. Conclusion

In this work, we have extended the work in Sussillo et al.(2007) by mathematically deriving a characterization of the STP parameter space that defines the transition between facilitating and depressing synaptic dynamics. Our result is only dependent on the form of the STP model used (Tsodyks et al., 1998), and is generally applicable to both spiking networks and rate based networks. For instance, our characterization implies the simple volumes used in Sussillo et al.(2007), as described in Section 3.2.1 and Figure 3, and simplifies the application of their techniques in a general setting. Furthermore, the transition from facilitating to depressing dynamics predicted by *r*_crit_ can be experimentally verified, potentially leading to refinements of the underlying STP model.

Finally, through simulation, we studied the problem of estimating STP parameters that can induce SS-RAIN network activity. We found that using an analytical framework characterized by *r*_crit_ increases the likelihood of finding self-sustaining networks by three orders of magnitude over a random search of the STP parameter space. This experiment was intended as a specific application of the analytical STP characterization derived in Section 3.1 and not intended as a thorough study of SS-RAIN. Though a thorough study of SS-RAIN coupled with STP in the spirit of Kumar et al.(2008) would be interesting, it is beyond the scope of this work. Future studies will be required to fully understand how STP affects SS-RAIN, including variations of network scale and connectivity.

### Conflict of interest statement

The authors declare that the research was conducted in the absence of any commercial or financial relationships that could be construed as a potential conflict of interest.

## Appendix

### Linear perturbation analysis

Here we expand upon the arguments of Sussillo et al.(2007). The following assumes a steady state, described in Equations (9), (11) and (12). As noted in Section 2.2, we use {*U*, *D*, *F*} in the following analysis.

We consider a network of two populations: excitatory and inhibitory. Let *W_mn_* be the mean synaptic weight from the *n* population to the *m* population, where *m*, *n* ∈ {exc, inh} are generic subscript labels. When specificity is required, we reserve the labels *e* = exc and *i* = inh. Let μ_*mn*_ be the mean dynamic synapse between the populations. We assume that the dynamic synapses are instantly equilibrating functions of the presynaptic firing rates. We assume that the synaptic weights *W_mn_* are chosen to produce a stable network firing rate column vector **r**^*^ = (*r*^*^_*e*_, *r*^*^_*i*_)^*T*^ for a constant current input column vector **v** = (*v_e_*, *v_i_*)^T^. Here, *r_e_* and *r_i_* are the average firing rates of the excitatory and inhibitory populations and *v_e_* and *v_i_* are the external inputs to excitatory and inhibitory populations. Recall that for **r**^*^, we have μ_*mn*_ = *W_mn_*, from the choice of the constants *A_ij_* in Equation (13). Further, we assume that for some decay constant τ_*m*_,

(A1) $$\tau_{m}\frac{dr_{m}}{dt}=-r_{m}+f_{m}[v_{m}+n_{e}{\bar{\mu }}_{me}(r_{e})r_{e}-n_{i}{\bar{\mu }}_{mi}(r_{i})r_{i}],$$

where *f_m_* is some monotonic function, called the *rate transfer function* for *n_e_* and *n_i_* presynaptic excitatory and inhibitory connections, respectively. This is an approximation to the network dynamics, commonly referred to as the *mean field* approximation (Van Vreeswijk and Sompolinsky, 1996, 1998; Tsodyks et al., 1998). For a constant network firing rate **r**^*^, Equation (A1) yields

(A2) $$r_{m}^{*}=f_{m}[v_{m}+n_{e}{\bar{\mu }}_{me}^{*}(r_{e}^{*})r_{e}^{*}-n_{i}{\bar{\mu }}_{mi}^{*}(r_{i}^{*})r_{i}^{*}].$$

We use perturbation theory to examine the change in firing rate **r**^*^ + δ**r** = (*r*^*^_*e*_ + δ *r_e_*, *r*^*^_*i*_ + δ *r_i_*)^*T*^ for a change in external input **v** + δ**v** = (*v_e_* + δ*v_e_*, *v_i_* + δ*v_i_*)^*T*^. With this perturbation, and the instant equilibration of the dynamic synapses, A2 becomes

(A3) $$\begin{array}{l} r_{m}^{*}+\delta r_{m}=f_{m}[\;v_{m}+\delta v_{m}\\ \;+n_{e}{\bar{\mu }}_{me}^{*}(r_{e}^{*}+\delta r_{e})(r_{e}^{*}+\delta r_{e})\\ \;-n_{i}{\bar{\mu }}_{mi}^{*}(r_{i}^{*}+\delta r_{i})(r_{i}^{*}+\delta r_{i})\;]. \end{array}$$

For *z* = *F*(*x*), we use the linearization δ*z* ≈ *F*′(*x*) · δ*x*. Up to first order in δ**r**, Equation (A3) becomes

(A4) $$\begin{array}{l} \delta r_{m}\approx \beta_{m}(\delta v_{m}+{\bar{W}}_{me}\delta r_{e}+d_{me}\delta r_{e}r_{e}^{*}\\ \;-{\bar{W}}_{mi}\delta r_{i}-d_{mi}\delta r_{i}r_{i}^{*}), \end{array}$$

where $d_{mn}=\frac{d{\bar{\mu }}_{mn}}{dr_{n}^{*}}$, β_*m*_ = *f*′_*m*_(*v_m_* + *W_me_r*^*^_*e*_ − *W_mi_r*^*^*_i_*), and we have used that μ^*^_*mn*_(*r*^*^_*n*_) = *W_mn_*.

Define the following matrices:

$$W:=B\left(\begin{array}{c} {\bar{W}}_{ee}-{\bar{W}}_{ei}\\ {\bar{W}}_{ie}-{\bar{W}}_{ii} \end{array}\right)N,\;D:=B\left(\begin{array}{c} d_{ee}-d_{ei}\\ d_{ie}-d_{ii} \end{array}\right)\left(\begin{array}{c} r_{e}^{*}\;0\\ 0\;r_{i}^{*} \end{array}\right)N,$$

where **B** and **N** are defined as

$$B:=\left(\begin{array}{c} \beta_{e}\;0\\ 0\;\beta_{i} \end{array}\right),\;N:=\left(\begin{array}{c} n_{e}\;0\\ 0\;n_{i} \end{array}\right).$$

With this notation, Equation (A4) can be written as

(A5) δr≈Bδv+Wδr+Dδr.

The solution to this system is

(A6) $$\delta r\approx {\left(I-(W+D)\right)}^{-1}B\delta v,$$

where *I* is the identity matrix. We proceed with the assumption that the dynamic synapses are almost static with the derivative matrix **D** ≈ **0**. We approximate δ**r** with respect to δ**v** up to  (**D**^2^).

In the following, we refer to the components of a matrix **M** with subscripts, as in **M**_ℓ*k*_. We invert the matrix in Equation (A6), while ignoring higher order terms from **D**, and we get

(A7) $$\begin{array}{l} \delta r\approx \frac{1}{\alpha -c}\left(\begin{array}{c} 1-W_{22}-D_{22}\;W_{12}+D_{12}\\ W_{21}+D_{21}\;1-W_{11}-D_{11} \end{array}\right)\left(\begin{array}{c} \beta_{e}\;0\\ 0\;\beta_{i} \end{array}\right)\delta v\\ \;=\text{​}\frac{1}{\alpha -c}\left(\begin{array}{c} \beta_{e}\delta v_{e}\left(1-W_{22}-D_{22}\right)+\beta_{i}\delta v_{i}\left(W_{12}+D_{12}\right)\\ \beta_{e}\delta v_{e}\left(W_{21}+D_{21}\right)+\beta_{i}\delta v_{i}\left(1-W_{11}-D_{11}\right) \end{array}\right), \end{array}$$

where we have defined

(A8) $$\alpha :=(1-W_{11})(1-W_{22})-W_{12}W_{21}$$

and

(A9) $$\begin{array}{l} c:=D_{11}(1-W_{22})+D_{22}(1-W_{11})\\ \;+W_{12}D_{21}+W_{21}D_{12}. \end{array}$$

Note that *c* = (**D**). We now estimate $\frac{1}{\alpha -c}$ with respect to assumptions that **D**^2^ ≈ **0** and sup **D**_ℓ*k*_ ≪ inf **W**_ℓ*k*_, which implies that $\frac{c}{\alpha }<1$, since every term of *c* contains an element of **D**. We get:

Combining Equations (A7) and (A10), we can estimate the change in excitatory firing rate with respect to the change in excitatory input:

(A10) $$\begin{array}{l} \frac{\delta r_{e}}{\delta v_{e}}\approx \frac{\beta_{e}}{\alpha }\left(1+\frac{c}{\alpha }\right)\left(1-W_{22}-D_{22}\right)\\ \;=\frac{\beta_{e}}{\alpha }\left[(1-W_{22})\left(1+\frac{c}{\alpha }\right)-D_{22}\left(1+\frac{c}{\alpha }\right)\right]\\ \;\approx \frac{\beta_{e}}{\alpha }\left[(1-W_{22})\left(1+\frac{c}{\alpha }\right)-D_{22}\right]\\ \;=\frac{\beta_{e}(1-W_{22})}{\alpha }\left(1+\frac{c}{\alpha }-\frac{D_{22}}{1-W_{22}}\right)\\ \;=\frac{\beta_{e}(1-W_{22})}{\alpha }(1+\frac{D_{11}(1-W_{22})}{\alpha }+\frac{D_{22}(1-W_{11})}{\alpha }\\ \;+\frac{W_{12}D_{21}}{\alpha }+\frac{W_{21}D_{12}}{\alpha }-\frac{D_{22}}{1-W_{22}})\\ \;=\frac{\beta_{e}(1-W_{22})}{\alpha }(1+\frac{D_{11}(1-W_{22})}{\alpha }+\frac{W_{12}D_{21}}{\alpha }+\frac{W_{21}D_{12}}{\alpha }\\ \;+\frac{D_{22}\left[(1-W_{11})(1-W_{22})-\alpha \right]}{\alpha (1-W_{22})})\\ \;=\frac{\beta_{e}(1-W_{22})}{\alpha }(1+\frac{D_{11}(1-W_{22})}{\alpha }+\frac{W_{12}D_{21}}{\alpha }+\frac{W_{21}D_{12}}{\alpha }\\ \;+\frac{D_{22}W_{12}W_{21}}{\alpha (1-W_{22})}). \end{array}$$

Substituting the values for the matrix components, we arrive at

(A11) $$\begin{array}{l} \frac{\delta r_{e}}{\delta v_{e}}\approx \frac{\beta_{e}(1+\beta_{i}n_{i}{\bar{W}}_{ii})}{\alpha }[1+d_{ee}\frac{\beta_{e}n_{e}r_{e}^{*}(1+\beta_{i}n_{i}{\bar{W}}_{ii})}{\alpha }\\ \;-d_{ie}\frac{\beta_{e}\beta_{i}n_{i}n_{e}r_{e}^{*}{\bar{W}}_{ei}}{\alpha }\\ \;-d_{ei}\frac{\beta_{i}\beta_{e}n_{e}n_{i}r_{i}^{*}{\bar{W}}_{ie}}{\alpha }\\ \;+d_{ii}\frac{\beta_{i}^{2}\beta_{e}n_{i}^{2}n_{e}r_{i}^{*}{\bar{W}}_{ei}{\bar{W}}_{ie}}{\alpha (1+\beta_{i}n_{i}{\bar{W}}_{ii})}]. \end{array}$$

Similarly, we get

(A12) $$\begin{array}{l} \frac{\delta r_{e}}{\delta v_{i}}\approx -\frac{\beta_{i}\beta_{e}n_{i}{\bar{W}}_{ei}}{\alpha }[1+d_{ee}\frac{\beta_{e}n_{e}r_{e}^{*}(1+\beta_{i}n_{i}{\bar{W}}_{ii})}{\alpha }\\ \;-d_{ii}\frac{\beta_{i}n_{i}r_{i}^{*}(1-\beta_{e}n_{e}{\bar{W}}_{ee})}{\alpha }\\ \;-d_{ie}\frac{\beta_{e}\beta_{i}n_{i}n_{e}r_{e}^{*}{\bar{W}}_{ei}}{\alpha }\\ \;+d_{ei}\frac{r_{i}^{*}(1+\beta_{i}n_{i}{\bar{W}}_{ii})(1-\beta_{e}n_{e}{\bar{W}}_{ee})}{\alpha {\bar{W}}_{ei}}]. \end{array}$$

We now prove that α is positive. If the synapses are static (**D** = **0**), then Equation(A6), reduces to

(A13) $$\delta r\approx {\left(I-W\right)}^{-1}B\delta v,.$$

Observe that α = det(**I** − **W**), and we have

(A14) $$\delta r\approx \frac{1}{\alpha }\left(\begin{array}{cc} 1-{\bar{W}}_{ii} & {\bar{W}}_{ei}\\ {\bar{W}}_{ie} & 1-{\bar{W}}_{ee} \end{array}\right)B\delta v.$$

Substituting in the appropriate values, and solving for the change in δ*r_e_* with respect to δ*v_e_*, we have

(A15) $$\frac{\delta r_{e}}{\delta v_{e}}=\frac{\beta_{e}}{\alpha }(1+\beta_{i}n_{i}{\bar{W}}_{ii}).$$

This can also be derived from Equation (A11) when all elements from **D** are set to zero. Because of the monotonicity of the rate transfer function, *f*, an increase to the excitatory inputs *v_e_* must result in an increase in *r_e_*, thus α > 0.

Observe also that **B** is positive-semidefinite by the monotonicity of *f*. With both α > 0 and **B** positive semi-definite, and all of the weights *W_mn_* ∈ (0, 1) are positive, the amount of change in Equation (A11) are determined by the signs of the derivatives *d_mn_* as discussed in Section 3.1.2.
